# Bone mineral density and diet of teachers of College of Home Economics at Lahore

**DOI:** 10.12669/pjms.314.8433

**Published:** 2015

**Authors:** Zahra Javed, Sardar Fakhar Imam, Neelam Imam, Kanwal Saba, Mulazim Hussain Bukhari

**Affiliations:** 1 Zahra Javed, Department of Food and Nutrition, College of Home Economics, Lahore – Pakistan; 2 Sardar Fakhar Imam, Department of Medicine, Fatima Jinnah Medical University, Lahore – Pakistan; 3 Neelam Imam, Department of Food and Nutrition, College of Home Economics, Lahore – Pakistan; 4 Kanwal Saba Department of Pathology Fatima Jinnah Medical University, Lahore – Pakistan; 5 Mulazim Hussain Bukhari Department of Pathology, Fatima Jinnah Medical University, Lahore – Pakistan

**Keywords:** Vitamin D, BMD, Osteoporosis, Balance Diet, Bone Pathology

## Abstract

**Objective:**

To evaluate the Bone Mineral Density (BMD) and diet of teachers of a Govt. College of Home Economics in Lahore.

**Methods:**

It was survey research. Purposive sampling technique was adopted for the selection of 50 teachers from Govt. College of Home Economics of age group 30 – 60 years.

**Results:**

About 46% of the subjects had BMD ratio in between -2.58 to -4.0 (Osteoporotic category). The root cause of low BMD ratio was not really age related but in majority of the sample it was due to sedentary life style and lack of awareness about the importance of exercise in relation to bone health.

**Conclusion:**

The total mineral and vitamin intake required for bone health (calcium, magnesium, phosphorus & vitamin D) was below the recommended, among majority of the sample.

## INTRODUCTION

Bones give structure to the body, help in moving and protecting our internal organs. Bones are made up of complex mixture of minerals (calcium, phosphate, magnesium) and various kinds of collagen and non-collagen proteins. Bone is a living tissue that changes constantly; the old bones are removed and replaced by new ones.^1^-^3^

Throughout life, bones change in size, shape, and position. Two processes guide these changes, modeling and remodeling. Bone is formed at one site and broken down in a different site.. However, much of the cellular activity in a bone consists of removal and replacement at the same site.^4^

Bone health is related not to calcium, phosphate, and Vitamin D essential for normal bone structure and function, but several other micronutrients and proteins also have essential roles in the development and maintenance of bone. Once the peak bone mass is achieved, it is important to maintain the bone mass gained and reduce the loss. This is possible by adopting a correct behavior of eating associated with regular physical activity and healthy lifestyle.^5^,^6^ Risk factors for osteoporosis include aging, being female, low body weight, low sex hormones such as during menopause, smoking and medication. The bone mineral density (BMD) is tested to determine the bone loss at an early stage.^7^,^8^

The aim of the present study was to evaluate bone mineral density and diet of teachers of College of Home Economics and to compare the diet of the women having low bone mineral density and high bone mineral density with standard bone health.

## METHODS

A total number of 50 teachers of age group 30–60 years were included in the study. Permission was taken from college for conducting this research. A pretesting was carried out before the actual research survey. Teachers from each department of college were selected for this pretesting. The survey tool was improved and finalized in the light of understanding gained from the pretesting. Data collection for research started from November 2013 to February 2014. Consent of the teachers was taken and an introduction about Bone Mineral Density and summary of research was given to the respondents. Interview schedule was used for collecting the desired information from the selected sample and it included a frequency checklist of all food groups, 24 hours recall along with BMD tests.

Dietary Record was taken by knowing food frequency checklist in 24 hours Recall. It included a bone mineral density (BMD) test to measure how much calcium and other types of minerals are in an area of a bone. This test helps detect osteoporosis and predicts the risk of bone fractures.

Anthropometric measurements including height and weight were recorded in every participant and body mass index (BMI) was calculated using the formula Weight in kg/height in m2.^9^

Statistical analysis was done using SPSS version 16. The data from 24 hour dietary recalls was used to assess the average (mean) intake of food from all food groups by the teachers. The mean intake of diet and its relation with BMD was then compared with the dietary reference intake. Continuous variables were expressed as mean±SD.

## RESULTS

About 12% of the subjects were between 30-40 years of age, 34% between 41-50 years and 54% were 51-60 years. The mean age was 45± 15.45 years. The 6% of the sample was underweight, 42% was of normal weight, and 38% was overweight, whereas 14% was obese. The 34% of the sample was having normal BMD ratio, 46% have osteopenia and 20% was osteoporotic. A huge difference was seen in the food habits and only 20% were found weekly restaurant user. Only 10% peoples were junk food eaters. Table-I & II.

**Table-I:** Distribution of data for satisfaction with weight.

Consumption Trend (%)	Yes	No	Total
Reduction required in weight <5kg	40	60	100
Reduction required in weight <5kg	42	58	100
Intake of soft drinks per day >5kg	84	16	100
Experience of bone fracture as an adult	12	88	100
Experience of joint pain	32	68	100
Swelling in joints	32	68	100

**Table-II:** Amount of nutrients consumed.

	Protein (Grams)	Carbohydrates (Grams)	Calcium (milligrams)	Sodium (milligrams)
Normal	77.4	168.46	11.61	8.8
				
Osteopenia	77.85	191.98	12.43	8.34
Osteoporosis	54.91	124.5	9.13	8.73

The 32% of the sample was taking 1-2 cups of tea per day, 54% 2-3 cups, 10% 3-4 cups whereas 4% of the sample was taking 5 cups or more daily. The 44% of the sample was premenopausal, where as 56% was menopausal. The 44% of the sample was not doing any exercise, whereas 28% was doing exercise on daily basis.

The study showed that 22% of the sample spent less than 15 minutes for exercise, 28% spent 15-30 minutes. Whereas 6% spent 30-60 minutes for exercise daily. The 42% of the sample was taking calcium supplements, 24% was taking vitamin D supplements, whereas 34% was taking both the supplements.

The study also showed that the mean and Standard deviation of amount of protein, Carbohydrates, Calcium and Sodium consumed by teachers having normal BMD, Osteopenia and osteoporosis. The low BMD ratios wee not really age related. As in age group of 30-40 years the percentage of Osteopenic sample is the highest. Table-III. The teachers’ weekly consumption of different foods and the detail of food frequency is shown in Table-IV.

**Table-III:** Relationship of BMD with age.

	Normal	Osteopenia	Osteoporosis
30-40	33	50.00	16
41-50	41	41.00	17
>50	29	48.00	22

**Table-IV:** Weekly consumption of different foods.

Food	>7	7	6	5	4	3	2	1	0
Cereal	Whole wheat	22	2	4	6	4	-	-	2	60
	White flour	-	2	2	-	-	2	-	2	92
	Mixed flour	6	14	6	12	14	4	0	0	44
	Rice	0	4	2	6	10	36	38	2	2
	Paratha	0	0	0	0	0	0	6	4	88
	Pasta	0	0	0	0	0	0	10	2	88
Meat & Meat products	Chicken	0	6	4	24	24	38	0	0	4
	Beef	0	0	0	0	0	0	32	40	54
	Mutton	0	0	0	0	6	40	36	12	6
	Fish	0	0	0	0	2	6	18	8	66
Eggs	Boiled egg	0	0	2	4	8	10	22	6	48
	Fried egg	0	4	2	8	18	24	14	4	26
	Scrambled egg	0	0	0	0	0	0	16	8	76
Milk & Milk Products	Whole milk	28	20	4	20	0	10	12	0	6
	Skimmed milk	8	4	4	4	2	4	2	2	70
	Yoghurt	2	0	4	22	24	26	16	4	2
	Cheese	0	0	0	0	0	4	24	10	62
	Ice cream	0	0	0	0	2	6	20	24	48
Vegetables and Fruits	Deep yellow vegetable	0	2	0	6	14	36	26	10	6
	Dark green leafy vegetable	0	2	0	6	6	30	44	6	6
	Starchy vegetable	0	2	0	4	4	46	36	2	6
	Fresh fruits	8	28	16	26	16	2	2	2	0
	Canned fruits	0	0	0	0	4	4	22	14	55
	Citrus fruits	2	0	0	22	24	10	24	0	8
	Other (dried/roasted)	2	4	2	6	20	32	18	6	10

## DISCUSSION

The study showed that 29% had normal BMD, 48% had osteopenia and 225 had osteoporosis. About 12% of the sample was of age group 30-40 years with 33% normal BMD, 50% osteopenic and 16% osteoporotic. Numerous cross-sectional and longitudinal studies using bone mineral density (BMD) by dual energy x-ray absorptiometry (DXA), depict the overall pattern of bone loss in both sexes.^10^,^11^

The teachers were not satisfied with their weight, 40% wanted to reduce at least 5 kg and 42% wanted to reduce more than 5 kg. A study was undertaken to evaluate the effect of obesity on the postmenopausal bone mass. The 176 women aged 45-71 years were divided into four groups according to their menopausal status and their weight: 49 premenopausal, 28 obese premenopausal, 49 obese postmenopausal. Comparison between groups revealed a significant effect of menopausal status and obesity on BMD and bone turnover. In premenopausal women, BMD was low, osteocalcin and calcium: Creatinine was higher only in non-obese postmenopausal women. The results of this study suggest that even moderate obesity can play a protective role on postmenopausal bone loss.^12^

We found that 34% of the total sample was having normal BMD ratio, 46% was osteopenic and 20% was osteoporotic. This indicate that majority of the sample was having weak bones. These findings are consistent with other studies.^7^,^12^

Almost 52% of the subjects were visiting restaurants once per month and their food preferences while eating out was Chinese (32%), junk food (10%) and (29%) Desi food. Majority of the subjects (86%) was taking one cup of milk per day which is not enough to fulfill the requirements. Calcium rich diet is essential for bone health. Findings from a Chinese study has suggested that an intake exceeding 900mg calcium/day was helpful in prevention of cortical bone loss among early postmenopausal Chinese women.^13^

Majority of the sample was consuming 1-2 servings of fruits and vegetables daily. Alkali provision may explain why fruits and vegetables benefit bone heath. A randomized placebo-controlled trial was conducted in 276 post postmenopausal women (aged 55-65 years) to determine the effect of alkali-providing potassium citrate and fruit and vegetable intake on bone turn over for 2 years.^14^

Another clinical trial indicated that the consumption of dried plum daily by postmenopausal women significantly increased serum marker of bone formation, total alkaline phosphatase, bone-specific alkaline phosphatase and insulin-like growth factor-I by 12, 6, and 17% respectively.^15^

This study showed that 84% of the subjects were consuming soft drinks and 12% was consuming 500-750 ml (2-3 glasses) of soft drinks per day which is not safe for health. Almost 64% of the subjects were taking 2-4 cups whereas 4% of the subjects were taking more than five cups daily. It has been demonstrated that caffeine increases urinary excretion of calcium, magnesium, sodium, chloride and water in at least three hours after intake. The increased losses are likely to be attributed to a reduction in renal reabsorption. While adaptation to most of the stimulating effects of caffeine occurs after a couple of day’s intake, there is no apparent adaptation to caffeine induced hypercalciuria.

The 44% of the sample was premenopausal, whereas 56% was menopausal. Estrogen is a female hormone that plays an important role in the health of women. One of its benefits is that it protects bones and helps keep them strong and healthy. When estrogen levels drop, many women lose bone density. As a result, their bones may not be as strong. For midlife women, the drop in estrogen that happens with menopause can lead to rapid bone loss. As a woman nears menopause, her monthly periods become less regular and can be heavy or light, or both at different times.^16^

Majority of the subjects 72% experienced joint pains, whereas 32% of the sample experienced swelling in joints. About 42% of the subjects were taking calcium supplements and 24% was taking Vitamin D supplements, whereas 34% was taking both the supplements. Combined calcium and vitamin D supplementation leads to reduction in non-vertebral fracture among elderly women. Production of Vitamin D by the skin declines with aging, which affects the absorption of calcium.^17^

We also found that age is not the only factor which affects the BMD ratios, because sample of age group 30-40 years has greater percentage of Osteopenia. Majority of the subjects was consuming whole wheat chapatti (22%), 36% of the sample was taking rice 3 times in a week. White flour bread was used by the majority (10%) >7 times in a week. It has been suggested that the ketoacidosis generated by low-carbohydrate/high-protein diets results in hypercalcuria. In turn, the skeleton supplies serum buffer by active resorption of bone, thereby leading to hypercalciuria and an adverse effect on bone health.^18^

We also observed that the consumption of chicken was most among this food group. Frequency of using chicken was much higher than mutton and beef. Intake of fried egg was higher as compared to boiled and scrambled egg. Weekly consumption of milk & milk products was there in the diet, but it needs to be improved. Whole milk and Yoghurt was consumed by majority of the subjects but it was considerably below the recommended dietary intake. Other milk products were used occasionally. Milk serves as an excellent source of calcium, with around 300milligrams per 8-ounce glass. To meet dietary calcium requirements, women need 1,000 milligrams per day between ages 19 and 50 and 1,200 milligrams after 51 years of age. Drinking three to four glasses of milk would meet one’s calcium needs.^19^

### Limitations of the study

We can’t generalize these findings because the female teachers may not be representative of the general population as they may have some unknown risk factors that are unique to them. Other limitations of the study include age of the teacher that majority might have not have reached peak BMD and small sample size in our study.

## CONCLUSION

Majority of the subjects was having weak bones. It was seen that the root cause of low BMD ratio was not really age related but in majority of the sample it was due to sedentary life style and lack of awareness about the importance of exercise in relation to bone health. It was also observed that the total mineral and vitamin intake required for bone health (calcium, magnesium, phosphorus & vitamin D) was below the recommended intake, among majority of the sample.

## RECOMMENDATIONS

Nutrition education for creating awareness about the importance of balanced diet including adequate calcium intake for attaining healthy bone should be given to teachers as well as to students, along with awareness about the importance of exercise in relation to bone health.
